# Protocol for PIT: a phase III trial of prophylactic irradiation of tracts in patients with malignant pleural mesothelioma following invasive chest wall intervention

**DOI:** 10.1136/bmjopen-2015-010589

**Published:** 2016-01-27

**Authors:** N Bayman, D Ardron, L Ashcroft, D R Baldwin, R Booton, L Darlison, J G Edwards, L Lang-Lazdunski, J F Lester, M Peake, R C Rintoul, M Snee, P Taylor, C Lunt, C Faivre-Finn

**Affiliations:** 1 Department of Clinical Oncology, The Christie NHS Foundation Trust, Manchester, UK; 2 The National Cancer Research Institute (NCRI) Consumer Liaison Group, London, UK; 3 Manchester Academic Health Science Centre Trials Co-ordination Unit (MAHSC-CTU), The Christie NHS Foundation Trust, Manchester, UK; 4 Respiratory Medicine Unit, David Evans Research Centre, Nottingham University Hospitals NHS Trust, Nottingham City Hospital Campus, Nottingham, UK; 5 Respiratory and Allergy Research Group, Institute of Inflammation & Repair, The University of Manchester North West Lung Centre, University Hospital of South Manchester NHS Foundation Trust, Manchester, UK; 6 Mesothelioma UK Charitable Trust, c/o Glenfield Hospital, Leicester, UK; 7 Department of Respiratory Medicine, University Hospitals of Leicester NHS Trust, Glenfield Hospital, Leicester, UK; 8 Department of Cardiothoracic Surgery, Chesterman Unit, Northern General Hospital, Sheffield Teaching Hospitals NHS Foundation Trust UK, Sheffield, UK; 9 Department of Thoracic Surgery, The Lister Hospital, London, UK; 10 Department of Clinical Oncology, Velindre NHS Trust UK, Cardiff, UK; 11 National Cancer Intelligence Network, (NCIN), Public Health England, London, UK; 12 Department of Thoracic Oncology, Papworth Hospital NHS Foundation Trust, Cambridge, UK,; 13 Department of Clinical Oncology, Leeds Teaching Hospital NHS Trust, St James Hospital, Leeds, UK; 14 Department of Medical Oncology, The Christie NHS Foundation Trust, Manchester, UK; 15 Department Pulmonary Oncology, Wythenshawe Hospital Manchester, Manchester, UK; 16 The University of Manchester, Manchester Academic Health Science Centre, Institute of Cancer Sciences, Manchester Cancer Research Centre (MCRC), Manchester, UK; 17 Radiotherapy Related Research, The Christie NHS Foundation Trust, Manchester, UK

## Abstract

**Introduction:**

Histological diagnosis of malignant mesothelioma requires an invasive procedure such as CT-guided needle biopsy, thoracoscopy, video-assisted thorascopic surgery (VATs) or thoracotomy. These invasive procedures encourage tumour cell seeding at the intervention site and patients can develop tumour nodules within the chest wall. In an effort to prevent nodules developing, it has been widespread practice across Europe to irradiate intervention sites postprocedure—a practice known as prophylactic irradiation of tracts (PIT). To date there has not been a suitably powered randomised trial to determine whether PIT is effective at reducing the risk of chest wall nodule development.

**Methods and analysis:**

In this multicentre phase III randomised controlled superiority trial, 374 patients who can receive radiotherapy within 42 days of a chest wall intervention will be randomised to receive PIT or no PIT. Patients will be randomised on a 1:1 basis. Radiotherapy in the PIT arm will be 21 Gy in three fractions. Subsequent chemotherapy is given at the clinicians’ discretion. A reduction in the incidence of chest wall nodules from 15% to 5% in favour of radiotherapy 6 months after randomisation would be clinically significant. All patients will be followed up for up to 2 years with monthly telephone contact and at least four outpatient visits in the first year.

**Ethics and dissemination:**

PIT was approved by NRES Committee North West—Greater Manchester West (REC reference 12/NW/0249) and recruitment is currently on-going, the last patient is expected to be randomised by the end of 2015. The analysis of the primary end point, incidence of chest wall nodules 6 months after randomisation, is expected to be published in 2016 in a peer reviewed journal and results will also be presented at scientific meetings and summary results published online. A follow-up analysis is expected to be published in 2018.

**Trial registration number:**

ISRCTN04240319; NCT01604005; Pre-results.

**Strengths and limitations of this study**

- Largest, adequately powered randomised controlled study of prophylactic radiotherapy in this population.
- Radiotherapy fields reflect current practice for thoracoscopy and drain insertion.
- Monthly telephone follow-up to supplement out-patient follow-up clinical significance of chest wall metastases measured with visual analogue scale pain score.
- Will not determine role of prophylactic radiotherapy at the site of an indwelling pleural catheter.

## Introduction

Malignant pleural mesothelioma (MPM) is almost exclusively linked to asbestos exposure with a latency period that is usually more than 30 years and prognosis is poor.^1–4^ The median survival in the UK is 8.8 months and the 1-year survival 38.5%.^5^

Diagnosis of mesothelioma is usually made by pleural biopsy either via CT-guided needle biopsy, thoracoscopy, video-assisted thoracic surgery (VATs) or thoracotomy. Patients often have associated pleural effusions and require chest drains to relieve symptoms such as chest pain and difficulty breathing. These invasive procedures encourage tumour cell seeding at the site of the intervention, which can result in formation of a subcutaneous tumour. The rate of chest wall metastases ranges from 2% to 50%, and depends on the procedure performed.^4^ ^6–14^

In an effort to minimise tumour seeding and prevent nodule development, it has been widespread practice for more than 20 years in the UK to irradiate intervention sites postprocedure—a practice known as prophylactic irradiation of tracts (PIT).

Only three randomised controlled trials, the largest with 61 patients, have assessed the role of PIT with conflicting results reported.^14–19^ One study reported a statistically significant reduction in the frequency of malignant seeding of tracts in the PIT arm compared to the control arm^14^; the other two studies did not.^15^ ^16^ Systematic reviews of the literature have concluded that the randomised trials conducted to date were considered the best available evidence for PIT, but that there was insufficient evidence to definitively recommend PIT.^17–20^ Inconsistencies are also evident in current national recommendations on the use of PIT. The British Thoracic Society^21^ recommends the use of PIT to prevent chest wall nodule formation following an interventional procedure. In contrast, the Cancer Care Ontario Programme^22^ stated that a recommendation for PIT in MPM could not be made due to inconsistent evidence, reflecting a lack of high-quality data. Similarly, in 2010 the European Respiratory Society stated “The value of prophylactic radiotherapy is questionable”^23^ and the European Society of Medical Oncology (ESMO) clinical recommendations for diagnosis, treatment and follow-up of MPM state that “it remains impossible to draw definitive conclusions regarding its efficacy”.^24^

Currently in the UK the routine use of PIT in patients with MPM depends on locality. A 2008 UK survey showed that 75% of radiotherapy centres are routinely using PIT.^17^ In addition, the small number of patients recruited to each arm, the high death rates, and overestimation of the rate of chest wall metastasis in the control arms, question whether the three previous randomised controlled trials were adequately powered. Furthermore, recent evidence shows that portal tracts are not always perpendicular to the skin^25^ and thus the small radiation fields centred on the chest wall scar employed by the previous negative studies may have been suboptimal.

There is now strong evidence to support the role of palliative chemotherapy in patients with MPM^26^ which is approved in the UK by the National Institute of Clinical Excellence (NICE).^27^ Importantly, the previous studies were conducted before palliative chemotherapy was widely used in MPM. Therefore, the role of PIT in the era of effective palliative chemotherapy remains undefined. In addition, no previous studies have used validated pain scoring tools to assess pain from tract metastases.

We believe a trial is essential for the following reasons: first an adequately powered RCT employing radiotherapy techniques ensuring adequate coverage of the entire portal tract will establish whether PIT should be offered routinely to patients with MPM after chest wall intervention in the era of palliative chemotherapy; second to establish whether chest wall metastases are symptomatic and thus, whether PIT, if effective, is clinically significant.

The hypothesis for this trial is that prophylactic irradiation of tracts will reduce the incidence of chest wall nodules from 15% to 5%. The primary objective of the PIT study is to determine the efficacy, as assessed by the incidence of chest wall metastasis, of prophylactic irradiation of tracts following invasive chest wall intervention in malignant pleural mesothelioma compared to no prophylactic radiotherapy. Secondary objectives include: the description of the toxicity of PIT; time from randomisation to chest wall tract metastasis in patients undergoing PIT compared with the no prophylactic radiotherapy; the assessment of pain from chest wall metastasis.

## Methods and analysis

### Trial design

**Figure 1:**
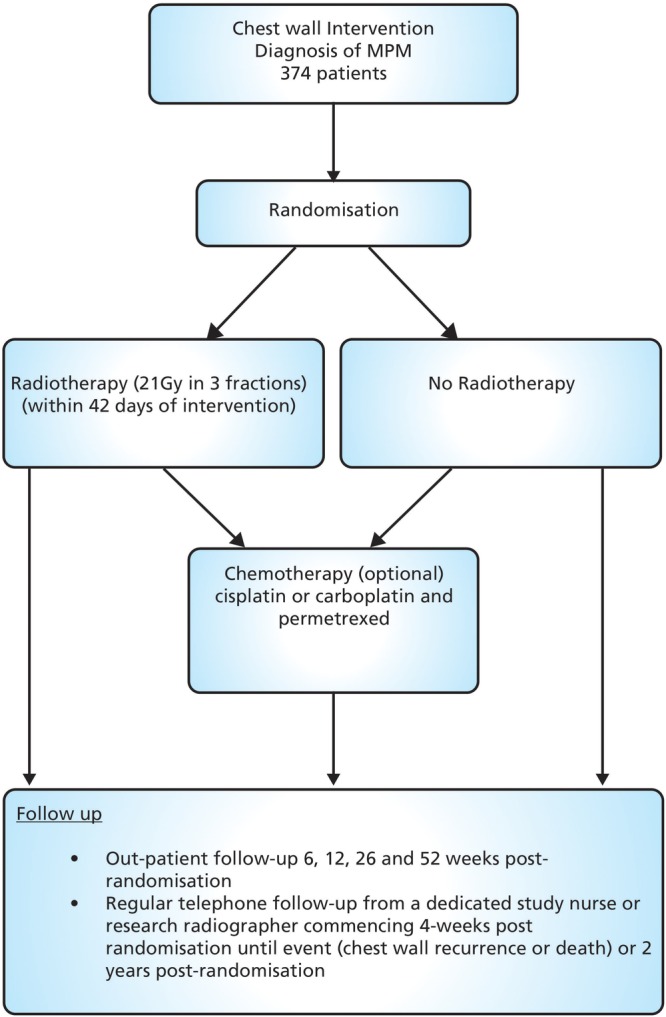
Trial schema/flow chart. MPM, malignant pleural mesothelioma.

This is a two-arm, phase III multicentre (list of centres can be found in online supplementary appendix 1 including a mix of academic centres and community hospitals) randomised superiority trial comparing radiotherapy versus no radiotherapy in patients with a histological diagnosis of MPM following chest wall intervention (see figure 1). A total of 374 patients will be randomised on a 1:1 basis to receive PIT (experimental arm) or no PIT (control arm). Patients can receive chemotherapy postradiotherapy/randomisation at the discretion of the treating physician.

The follow-up period for the study is up to 2 years postrandomisation which includes regular telephone follow-up and a minimum of four outpatient visits in the first year.

### Patient selection

Potential participants will be identified by the principal investigator and his/her team at each centre, via the discussion of cases in a multidisciplinary team meeting (MDT). If a patient is identified as a potential participant in the PIT trial they will be approached at their next clinic visit, as part of discussions with the patient about their options for treatment. Patients who are interested in receiving further information about the trial will be given a copy of the patient information sheet (see online supplementary appendix 2) and consent form (see online supplementary appendix 3), and will have the opportunity to discuss the trial in detail before deciding whether to participate.

### Inclusion criteria

- Either sex, age ≥18 years.
- Diagnosis of mesothelioma by MDT
- All histological subtypes. Where the histological diagnosis is unclear, a specialist thoracic pathologist should be consulted.
- Eastern Cooperative Oncology Group performance status 0–2.
- Inoperable disease or operable disease in patients unsuitable for surgery as decided by a MDT.
- Chest wall intervention with video-assisted thoracoscopy (VATS), open surgical biopsy, local anaesthetic thoracoscopy or chest drain.
- Able to start radiotherapy within 42 days (6 weeks) from most recent chest wall intervention.
- Chest wall intervention scar visible at time of randomisation.
- No indwelling pleural catheters in situ at the intervention site.
- Radiotherapy target volume acceptable by the local radiotherapist.
- No previous open thoracotomy.
- No previous radiotherapy to the region of the chest wall intervention site.
- Not currently receiving chemotherapy and not received chemotherapy for mesothelioma before randomisation.
- No other previous or concomitant illness or treatment which in the opinion of the clinician will interfere with the trial treatments or comparisons.
- Patients enrolled on other clinical trials could be considered after discussion with the chief investigators.
- Female patients must satisfy the investigator that they are not pregnant, or are not of childbearing potential, or are using adequate contraception.
- Patients must not be breastfeeding.
- Absence of any psychological, familial, sociological or geographical condition potentially hampering compliance with the trial protocol and follow-up schedule; those conditions should be discussed with the patient before randomisation in the trial
- Patients can only be randomised in this trial once.

### Exclusion criteria

- Patients who underwent a thoracotomy (as large thoracotomy scars may not be adequately covered by this radiotherapy technique).
- Previous radiotherapy to the region of the chest wall intervention site.
- Indwelling pleural catheter in situ at the intervention site.
- Patients currently receiving chemotherapy.

### Consent

All patients will be informed of the aims of the trial, the procedures and possible adverse effects, and the mechanism of treatment allocation. Patients will be informed as to the strict confidentiality of their patient data, but that their medical records may be reviewed for trial purposes by authorised individuals other than their treating physician.

It will be emphasised that the participation is voluntary and that the patient is allowed to decline further participation in the protocol whenever he/she wants. This will not prejudice the patient's subsequent care. Documented informed consent will be obtained by local principal investigators or a delegated member of staff for all patients included in the trial before they are registered or randomised in the trial in accordance with the national and local regulatory requirements.

### Randomisation procedure

Patients will be randomised by phone or fax on a 1:1 basis to one of two treatment arms. A variant of an adaptive biased coin randomisation method will be used to favour balance between treatment arms in the four strata formed from the following two factors:

- Histology (epithelioid or not epithelioid)
- Intention to give chemotherapy

Randomisation will be undertaken centrally by Manchester Academic Health Science Centre Clinical Trials Unit (MAHSC-CTU).

### Radiotherapy

Patients are treated on a linear accelerator using a single electron field. Treatment fields can be shaped using individualised lead cut-outs of the appropriate thickness. The total dose of radiotherapy in the PIT arm is 21 Gy in three fractions, once daily over three consecutive days. The patient can be treated supine, prone or in the lateral position depending on the position of the chest wall intervention site. To ensure >90% of the prescribed dose is delivered to the skin surface, 0.5 cm tissue-equivalent bolus can be applied to the whole treatment field. The field position for radiotherapy is recorded so that if the patient develops chest wall nodules it can be determined if the nodules are inside or outside the treatment field.

The clinical target volume (CTV) comprises the visible and palpable scar with a 1 cm margin of clinically normal tissue in the lateral, medial and inferior directions at the skin surface. The planning target volume (PTV) comprises the CTV with a 1 cm margin in all directions at the skin surface. The CTV to PTV expansion allows for set up errors, changes in the electron beam profile at depth and patient motion. The treatment field is defined by adding a further 1 cm margin in all directions to account for the beam penumbra.

**Figure 2:**
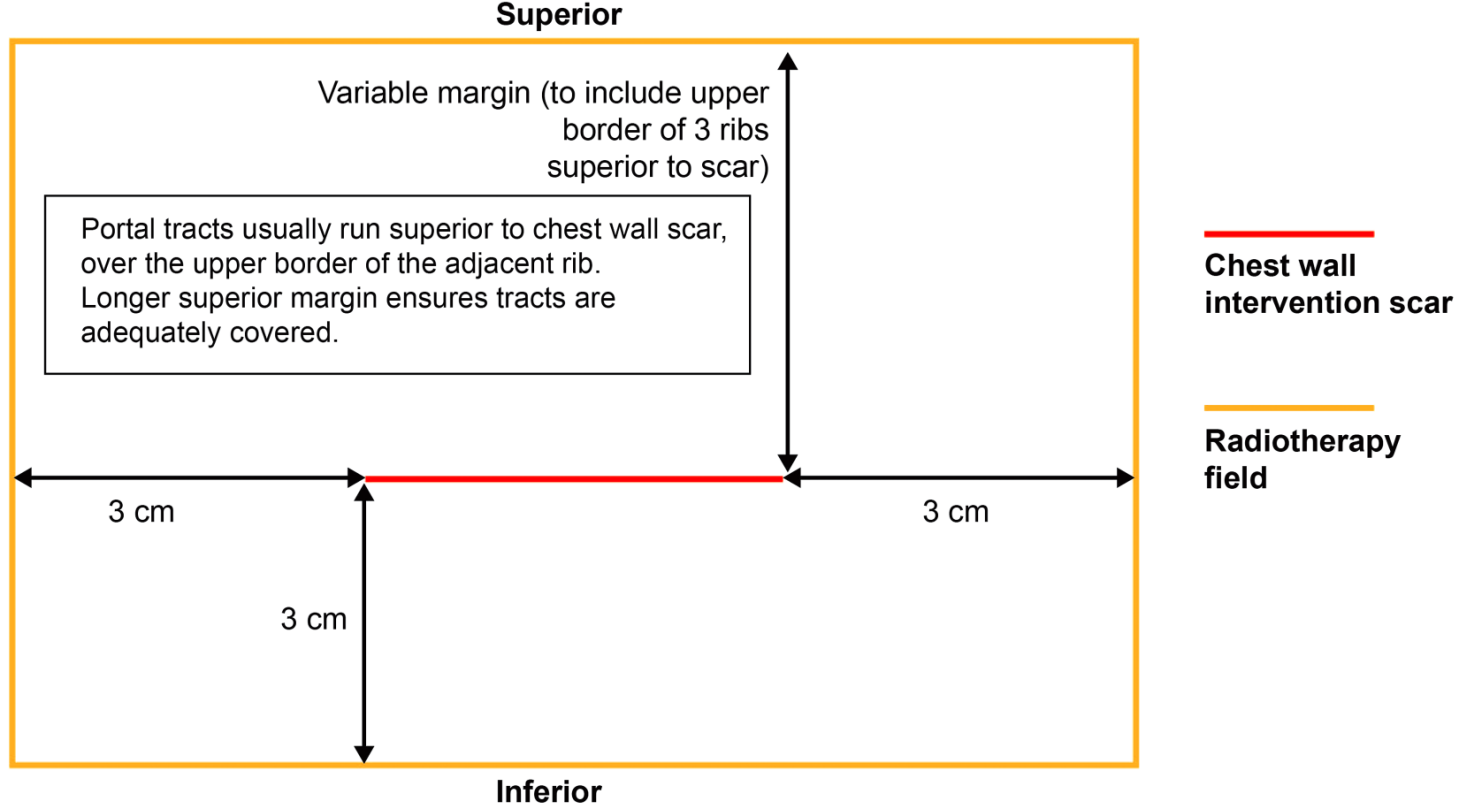
Prophylactic irradiation of tracts radiotherapy field margins.

The superior margin will vary and should be the determined by counting three ribs superiorly. The upper border of the third rib superior to the scar will be the superior border of the treatment field. This will take into account microscopic spread but also ensure that chest wall intervention tracts (which will commonly run over the rib superior to the scar) are covered at depth (see figure 2).

No organs at risk need to be identified and if a patient has two intervention sites, and therefore two scars (eg, a VATS scar and adjacent chest drain scar) these can be treated within a single treatment field or two separate fields. A gap of >1 cm must separate two treatment fields.

### Chemotherapy

Patients can be treated with palliative chemotherapy at the physician's discretion after completion of radiotherapy (experimental arm) or after randomisation (control arm). The efficacy and/or safety of chemotherapy is not being investigated as part of the study. The chemotherapy regimen used should be cisplatin or carboplatin plus pemetrexed, although alternative chemotherapy regimens (including clinical trial agents) may be considered after discussion with the chief investigators. The number of cycles given is left to the physician's discretion. A gap of at least 1-week must separate the completion of radiotherapy and the start of palliative chemotherapy. The chemotherapy regimen, start and stop dates and total number of cycles administered should be recorded. No further data needs to be collected concerning the patients chemotherapy treatment.

Concomitant medication-not applicable for this trial.

### Discontinuation or withdrawal of patients

In the case of disease progression, the patient will be treated according to each centre's policy. For patients allocated to the treatment arm, radiotherapy can be stopped at the participants request or if too unwell to continue at the discretion of the treating clinician.

### Outpatient visits

The patient is reviewed in clinic 6, 12, 26 and 52 weeks following randomisation to assess for any signs of chest wall metastasis and to record any treatment-related toxicities. In addition, telephone follow-up at 4-weekly intervals following randomisation will determine if the patient has noted any chest wall nodules. If a patient is suspicious that a nodule has appeared, they will be invited to clinic for assessment.

If a chest wall metastases is confirmed, the position of the metastases is recorded in relation to the reconstructed radiotherapy treatment field.

Patients are asked to complete a visual analogue score (VAS) for pain at baseline, and at each telephone and clinic follow-up. Patients are specifically asked to only consider pain at the original site of chest wall intervention. If the patient develops chest wall nodules they will be asked to complete a VAS for pain at the site of the chest wall intervention at for the three nodules closest to the original intervention.

### Outcome measures

The primary end point is the incidence of chest wall tract metastasis 6 months from randomisation. If a patient raises suspicion that a chest wall nodule had developed at telephone follow-up, they will be invited to attend an outpatient appointment with the investigator. If there is clinical evidence of a chest wall metastases, a chest wall metastasis cardio respiratory fitness (CRF) is completed using the clinic date as date of chest wall metastasis.

The secondary end points are:

- Time from randomisation to chest wall tract metastasis (recorded on chest wall metastasis CRF)
- Position of chest wall tract metastasis recurrence in relation to radiotherapy field in patients randomised to experimental arm (in field/out-of-field, recorded on chest wall metastasis CRF)
- Acute and late skin radiotherapy toxicity (recorded by CTCAE v4.0 at baseline and at each outpatient visit on CRF)
- Pain from chest wall tract metastasis (recorded by VAS pain scores recorded on chest wall metastasis CRF)

### Sample size calculation

The crude rate of tract metastases following chest wall intervention until death, based on historical data, is expected to be 15%. It is estimated that the majority of events will occur within 6 months of the intervention. A reduction in the incidence of chest wall nodules from 15% to 5% in favour of radiotherapy would be clinically significant. Based on a two-arm trial with a 5% significance level, two-sided test and 80% power this would require 280 patients. Furthermore it is anticipated that 25% of patients will not survive for 6 months therefore an additional 94 patients will be required, making the total number of patients to be enrolled 374.

### Analysis

#### Incidence of chest wall tract metastasis

All patients randomised will be analysed on an intention-to-treat basis. A two-sided χ^2^ test of proportions of chest wall tract metastasis at 6 months between the control and experimental arms will be used with a 5% significance level. Secondary analysis using logistic regression will be used to investigate treatment after adjusting for significant baseline prognostic variables.

#### Time to chest wall tract metastasis

Kaplan-Meier curves will be drawn for each treatment group. Time to chest wall tract metastasis will be compared using a two-sided log rank test with a 5% level of significance. Cox-proportional hazards models will be used to investigate the effect of treatment after adjusting for stratification factors and other significant baseline variables.

#### Toxicity

Skin toxicity will be assessed according to NCI Common Terminology Criteria for Adverse Events V.4.0. The proportions of patients experiencing a grade of 3 or above acute toxicity, including acute radiation morbidity, or late radiation morbidity will be compared between the treatment groups using a two-sided χ^2^ test with a 5% level of significance. Acute toxicity will be defined as toxicities occurring from start of treatment to 3 months after completion; late toxicity will be defined as toxicities occurring between 3 months and 2 years after completion of treatment.

#### Position of chest wall tract metastases

Position of the central point of a chest wall tract metastases in relation to the reconstructed radiotherapy treatment field will be recorded as in field/out-of field recurrence. Where the central point falls on the edge of the radiotherapy treatment field this will be recoded as in-field recurrence

#### Pain scores

Patients will be analysed using the intent-to-treat method. All tests will be two-sided and a p value of 0.05 or less will be considered statistically significant. In patients who develop tract metastasis, the VAS pain score from the assessment immediately before the occurrence of the tract metastasis will be considered baseline. Descriptive analysis will be performed to summarise change in pain score from baseline to subsequent pain score. Two-sample t tests will be used to explore difference between treatments. A 20% increase in VAS pain score is considered significant. The proportion of significant increases in pain in both arms will be analysed using a two-sided χ^2^ test.

No formal interim analysis is planned.

### Protocol adherence

No major problems are anticipated in terms of adherence to intervention protocols as the experimental arm only involves three fractions of standard radiotherapy delivered over approximately 5 min on three consecutive days.

### Data handling and analysis

All forms will be entered in a trial defined database for which some consistency checking will be programmed in. Data managers will check for missing and invalid data using SQL queries and statistical programs. Any queries will be highlighted on trial-specific query forms and returned to the centre for correction and/or clarification. The data will be stored on a secure server access to which is restricted to MAHSC-CTU staff. The data management procedures can be found in the PIT Data Management Manual, this is an internal document created for use by the Data Manager within the MAHSC-CTU and contains all procedures defined to ensure the data management and validation procedures are properly carried out.

## Ethics and dissemination

### Protocol and protocol amendments

The trial details documented here are consistent with PIT study protocol V.3.0 (dated: 19th April 2012, available on request from the trial manager, MAHSC-CTU).

There have been two substantial amendments for the study.

1. 25 April 2012—addition of an inclusion criterion ‘Indwelling pleural catheter in situ at the intervention site’ and several administrative updates to the protocol and patient information sheets. The amendment also included the home and outpatient VAS questionnaires.
2. 17 April 2013—introduction of the VAS chest wall nodule page which the patient is asked to complete if they develop chest wall nodules.

### Trial monitoring

The trial management group (TMG) is responsible for reviewing the trial's day-to-day activities, the overall supervision of the trial and ensure that it is being conducted in accordance with the principles of good clinical practice and relevant regulations. The group should agree any protocol amendments and provide advice to the investigators on all aspects of the trial. The TMG meets twice a year and includes patient/carer representatives, respiratory physicians, thoracic surgeons, clinical oncologists, medical oncologists and clinical nurse specialists. Three additional members who are independent of the trial have been appointed and will be available to advise the TMG should any issues arise requiring an independent viewpoint.

An Independent Data Monitoring Committee (IDMC) will not be appointed for this trial as PIT is currently widely used in clinical practice, it is not a new treatment and we not envisage that any safety issues will arise.

### Trial sponsorship

The study is sponsored by The Christie NHS Foundation Trust, Research & Development Department, Wilmslow Road, Manchester, M20 4BX, UK

### Trial management

The Manchester Academic Health Science Centre Clinical Trial Unit is co-ordinating the study. This includes data collection, management, monitoring, analysis and interpretation of data. The PIT TMG will write the report and will make the decision to submit the report for publication.

### Safety reporting

An adverse event (AE) is defined on this trial as any untoward medical occurrence in a clinical trial subject which does not necessarily have a causal relationship with the trial-related procedures. An AE can therefore be any unfavourable and unintended sign (including an abnormal laboratory finding), symptom or disease.

A serious adverse event (SAE) for this trial is an adverse event only if it meets the following criteria.

- Results in death (within 90 days of last dose of radiotherapy and is considered related to trial radiotherapy);
- Is life-threatening (and is considered related to the trial radiotherapy);
- Requires hospitalisation, or prolongation of existing hospitalisation (and is considered related to trial radiotherapy);
- Results in persistent or significant disability or incapacity (and is considered related to trial radiotherapy);
- Is a congenital anomaly or birth defect;
- Other medically significant event. Medical judgement should be exercised in deciding whether an AE is serious in other situations. Important AEs that are not immediately life-threatening or do not result in death or hospitalisation, but may jeopardise the subject or may require intervention to prevent one of the other outcomes listed in the definition above, should also be considered serious.

Disease progressions or events related to disease progressions are not considered to be SAEs. AEs relating to other anticancer treatments that the patient may be receiving are not considered to be SAEs.

All SAEs are reported by site teams to the MAHSC-CTU within 24 h of the investigator being made aware of the event.

### End of the trial

The study will close 2 years after the 374th patient is randomised. The Chief Investigator, TMG and/or the three independent members have the right at any time to terminate the study for clinical or administrative reasons. The end of the study will be reported to the REC and Regulatory Authority (where applicable) within the required timeframes.

Investigators will inform participants of any premature termination of the trial and ensure that the appropriate follow-up is arranged for all involved.

### Dissemination

Data from all centres will be analysed together and published as soon as possible. Individual participants may not publish data concerning their patients that are directly relevant to questions posed by the trial until the TMG has published its report. The TMG will have access to the final data set, form the basis of the Writing Committee and advise on the nature of publications. The trial will be publicised at regional and national meetings, and to patient groups with the support of Mesothelioma UK. The final results will be presented at scientific meetings and published in a peer-reviewed journal (authorship will be according to the journal's guidelines). A lay summary will be disseminated via local and national mesothelioma charities. Summary results will also be published online at clinicaltrials.gov and cancerhelp.
